# Wild Japanese Marten, *Martes melampus melampus* (Carnivora: Mustelidae), Is a New Host for Zoonotic *Thelazia callipaeda* Eyeworm

**DOI:** 10.1002/ece3.71439

**Published:** 2025-05-08

**Authors:** Toshihiro Tokiwa, Kandai Doi, Ayaka Kitajima

**Affiliations:** ^1^ Laboratory of Veterinary Parasitology Nippon Veterinary and Life Science University Musashino Tokyo Japan; ^2^ Forestry and Forest Products Research Institute Tsukuba Ibaraki Japan

**Keywords:** Japanese marten, oriental eyeworm, reservoir, sylvatic cycle, wildlife, zoonosis

## Abstract

*Thelazia callipaeda* is a spirurid nematode that parasitizes the eyes of mammals, including carnivores, lagomorphs, and humans. Although this parasite has been reported in domestic and wild animals in Japan, its presence in the Japanese marten, *Martes melampus melampus* (Carnivora: Mustelidae), has not been documented. In this study, we examined the ocular parasites of a wild Japanese marten collected in Tsukuba, Ibaraki Prefecture, Japan, and identified *T. callipaeda* based on morphological and molecular analyses. This is the first record of *T. callipaeda* infection in the Japanese marten. Our findings suggest that wild Japanese martens serve as a natural host for *T. callipaeda*, contributing to its transmission in the sylvatic cycle.

## Graphical abstract

Wild Japanese martens serve as a natural host for *Thelazia callipaeda*, contributing to its transmission in the sylvatic cycle.

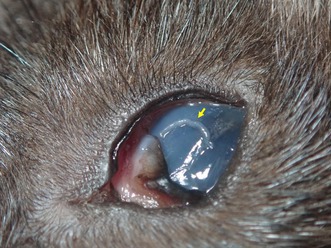

## 1 Introduction

*Thelazia callipaeda* Railliet and Henry, 1910 is an ocular nematode belonging to the family Thelaziidae, which parasitizes a wide range of mammalian hosts, including carnivores, lagomorphs, and humans (do Vale et al. 2020). Adult worms parasitize the conjunctival sac and lacrimal ducts, causing thelaziosis, which can lead to conjunctivitis, excessive lacrimation, and corneal ulcers in severe cases (Otranto et al. 2004). This nematode is transmitted by lachryphagous flies of the genus *Phortica* (Diptera: Drosophilidae), which serve as intermediate hosts. In Japan, thelaziosis has been reported in domestic animals such as dogs ( *Canis lupus familiaris* ) and cats ( *Felis catus* ), as well as in humans, mainly in the western part of the country. There has also been a single report of *T. callipaeda* in an imported European lynx ( *Lynx lynx* ) maintained in a zoo in Nagano, Japan (El‐Dakhly et al. 2012). However, recent studies have shown a northward expansion of the endemic area, with many animals serving as reservoirs for this parasite. These include the raccoon ( *Procyon lotor* , Procyonidae, non‐native), Japanese raccoon dog (*Nyctereutes viverrinus*, Canidae, native) (Doi et al. 2023), Japanese badger ( *Meles anakuma* , Mustelidae, native), Japanese black bear ( *Ursus thibetanus japonicus* , Ursidae, native), Japanese red fox ( *Vulpes vulpes japonica* , Canidae, native), and masked palm civet ( *Paguma larvata* , Viveriidae, non‐native) (Kitajima et al. 2024). Understanding the host range of *T. callipaeda* is important for evaluating its transmission dynamics and assessing transmission risk to domestic animals and humans.

In this study, we investigated an eyeworm infection in a wild Japanese marten ( *Martes melampus melampus* , Mustelidae), a medium‐sized mustelid native to Japan, and confirmed *T. callipaeda* infection using morphological and molecular analyses.

## 2 Materials and Methods

### 2.1 Specimens

**FIGURE 1:**
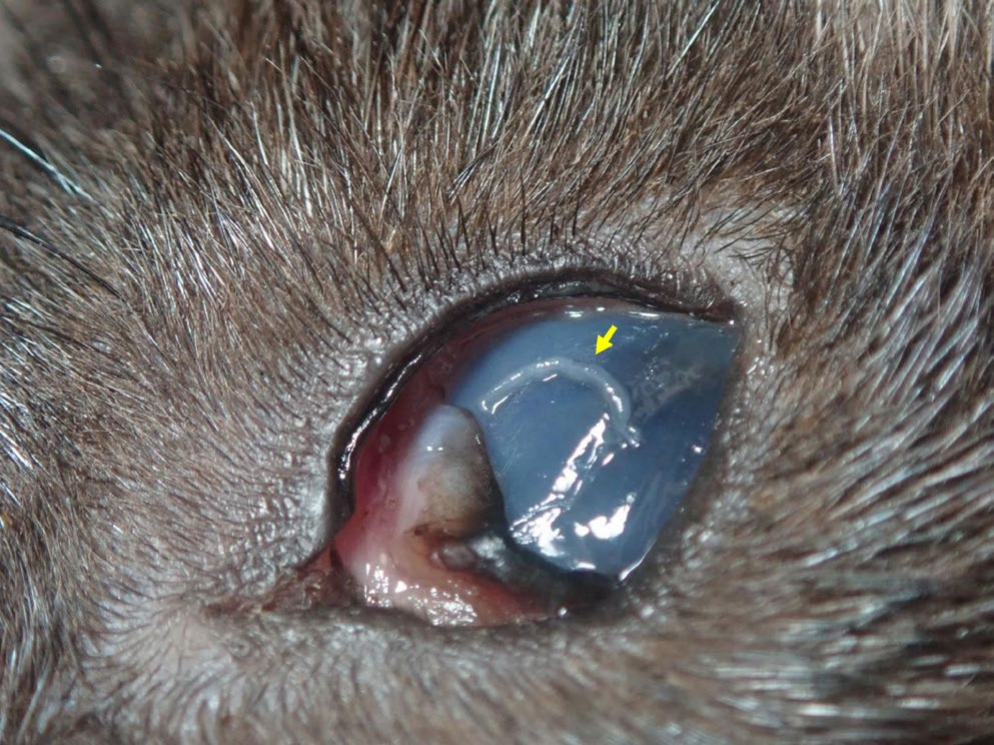
*Thelazia* eyeworm (arrow) on the conjunctiva of an adult male Japanese marten ( *Martes melampus melampus* ) in Ibaraki, Japan.

On December 11, 2024, a deceased adult male Japanese marten was found on a trail route on the Hokyo Mountain (36°16′15.70″ N, 140°13′25.60″ E) in Ibaraki Prefecture, Japan. The body was moderately decomposed. The weight postmortem was 1.25 kg, and the head‐to‐tail length was 67.7 cm. The worms were collected from the conjunctiva (Figure 1) and conjunctival sac of the eyes using swabs. The collected worms were preserved in 70% ethanol until analysis.

### 2.2 Morphological and Sequence Analyses

The eyeworms were permeabilized with a solution of glycerol (50%, *w/w*) and ethanol (40%, *w/w*), mounted on slides, and observed under a DM2500 microscope (Leica, Germany). The imaging and morphometry were performed using a Flexacam C5 (Leica). The nematode species was identified using identification keys provided by Faust (1928) and Otranto et al. (2003).

For genetic analysis, the middle portion of 10 worms was dissected out, and DNA was extracted using a DNA Mini Kit (Qiagen, Germany) according to the manufacturer's protocol. A partial sequence of the cytochrome *c* oxidase subunit 1 mitochondrial gene (*cox1*, 667‐bp) was amplified using the specific primers NTF (5′‐TGATTGGTGGTTTTGGTAA‐3′) and NTR (5′‐ATAAGTACGAGTATCAATATC‐3′) (Otranto et al. 2005). Polymerase chain reaction was performed using 20 μL reaction volumes, each containing 0.2 μL TaKaRa Ex Taq polymerase (TaKaRa, Japan), 1.6 μL dNTPs (2.5 mM of each), 2 μL of 10 × buffer, 1.0 μL template, and 14.8 μL double‐distilled water. Thermocycling was carried out with an initial denaturation at 95°C for 5 min, followed by 35 cycles of 94°C for 30 s, 50°C for 30 s, and 72°C for 1 min. A final extension was performed at 72°C for 10 min, and the reaction was then held at 4°C. PCR products were electrophoresed on 1.5% agarose gels, stained with GRGreen (BioCraft, Japan), and visualized using an LED transilluminator. Product sizes were determined by comparison with a 100‐bp DNA ladder (Maestrogen, Taiwan). The amplified products were purified using ExoSAP‐IT (Macrogen Corp., Japan) and subsequently sequenced on an ABI 3730xl DNA analyzer (Thermo Fisher Scientific, USA) with the same primers used for PCR. Multiple sequence alignment of the obtained *cox1* sequences and the *cox1* haplotype dataset reported in our previous study (Doi et al. 2023) was performed using the MAFFT online service ver. 7 (Katoh et al. 2019). The haplotype names are in accordance with previous reports (Otranto et al. 2005; Zhang et al. 2018; Doi et al. 2023).

## 3 Results and Discussion

**FIGURE 2:**
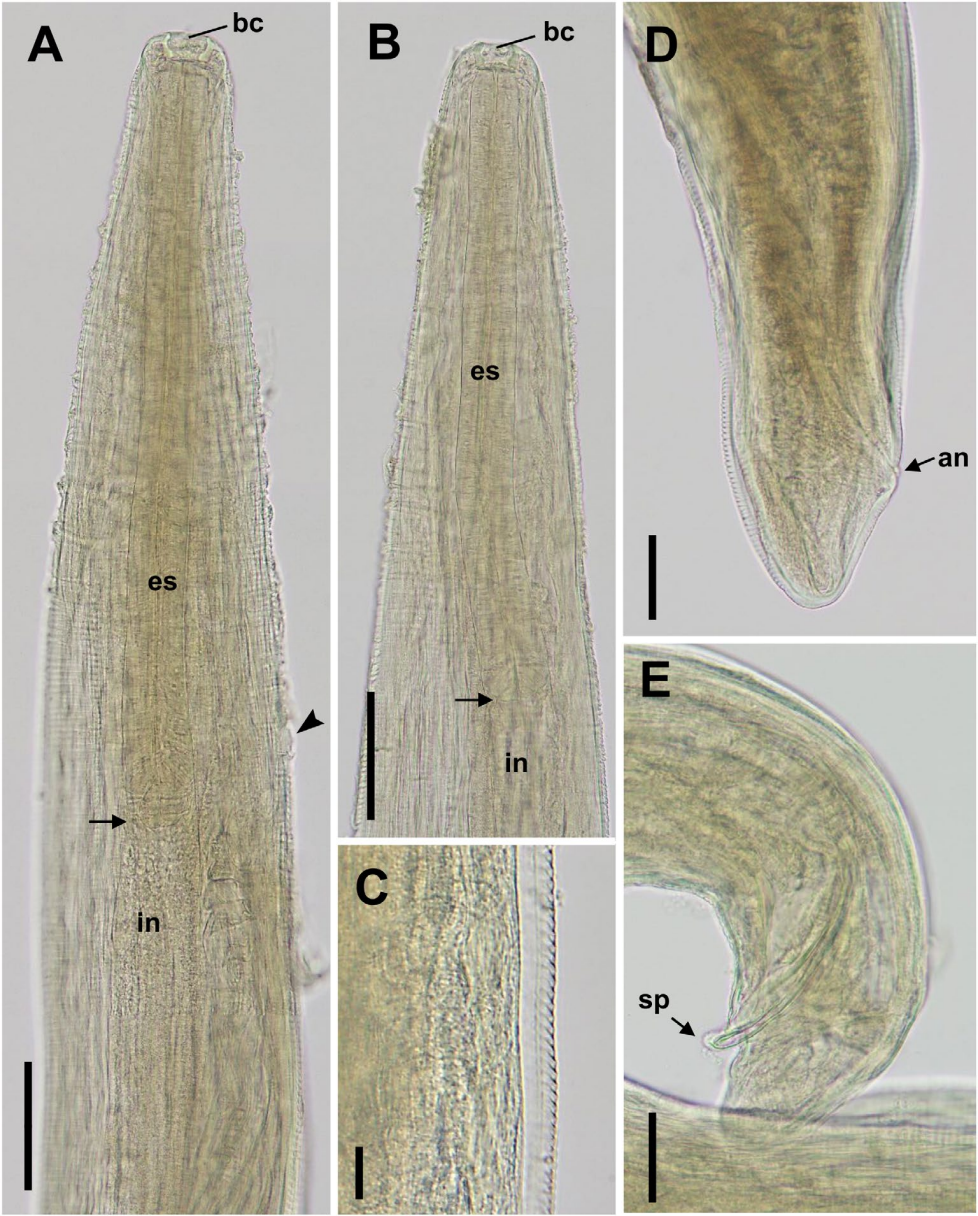
Microscopic images of *Thelazia* worms from the Japanese marten, *Martes melampus melampus* . (A) Anterior extremity of female, showing bowl‐shaped buccal capsule and vulva (arrowhead) located anterior to the esophago‐intestinal junction (arrow). Scale = 100 μm. (B) Anterior extremity of male. Scale = 100 μm. (C) Transversely serrated cuticle. Scale = 20 μm. (D) Posterior extremity of female. Scale = 50 μm. (E) Posterior extremity of male. Scale = 50 μm. an = anus; bu = buccal capsule; es = esophagus; in = intestine; sp = spicule.

The animal harbored a unilateral infection, with 11 worms (five females, three males, and three undetermined due to degeneration) recovered from the left eye. Morphological analysis of the worms indicated the presence of a cup‐shaped buccal capsule (Figure 2A,B), cuticular transverse striations (Figure 2C), and a short tail (Figure 2D,E). Female nematodes (*n* = 5) were 14.2–15.3 (mean = 14.8) mm long and 355–390 (370) μm wide; male worms (*n* = 3) were 8.2–11.2 (10.1) mm long and 326–380 (365) μm wide. In female worms, the vulva was located anterior to the esophago–intestinal junction (Figure 2A), and numerous larvae were present in the uterus. The number of cuticular transverse striations ranged from 300 to 400/mm in the head portion and 250 to 300/mm in the caudal region. Based on these characteristics, the worms collected from the Japanese marten were identified as *T. callipaeda*. Sequence analysis of the *cox1* from 10 worms of *T. callipaeda* from the Japanese marten indicated two distinct haplotypes. The first sequence, detected in six worms, was 100% identical to the *T. callipaeda* sequence representing the h9 haplotype, and the second sequence, found in four worms, was 100% identical to that of the h10 haplotype.

This is the first record of *T. callipaeda* in the Japanese marten, a medium‐sized mustelid found in a wide range of regions in Japan (Masuda 2015). The Japanese marten inhabits forested areas from the lowlands to the alpine regions, but can also be found in forest edges, grasslands, and agricultural lands (Masuda 2015). These habitats overlap with the distribution range of *Phortica* spp. (Okada 1988), suggesting that a sylvatic transmission cycle of *T. callipaeda* can occur. In Japan, the sylvatic transmission cycle is believed to involve animals such as Japanese black bears and Japanese red foxes (Kitajima et al. 2024). The urban or semi‐urban transmission cycle is thought to involve raccoon, Japanese raccoon dog, and masked palm civet, and the two haplotypes (h9 and h10) detected in this study appear to be shared among these animals. The co‐occurrence of these two haplotypes observed in the present case has likewise been reported in raccoon (Doi et al. 2023), masked palm civet, black bear, and Japanese red fox (Kitajima et al. 2024), suggesting their coexistence among wild animals in Japan. These two haplotypes are also responsible for thelaziosis in humans and domestic animals (Huang et al. 2024), indicating that *T. callipaeda* is distributed throughout Japan in various wild animals.

Table 1 summarizes the reports of *T. callipaeda* infections in mustelids, including the Japanese badger (Kitajima et al. 2024), European badger ( *Meles meles* ) (Ionică et al. 2019), sable ( *Martes zibellina* ) (Odoevskaya et al. 2015), and beech marten ( *Martes foina* ) (Otranto et al. 2009; Seixas et al. 2018; Ionică et al. 2019), making them the fifth host species in the family Mustelidae. This further indicates that *T. callipaeda* has broadened its host spectrum among mustelids. Mustelids include approximately 85 putative extant species found worldwide (Law et al. 2018) and include weasels (subfamilies Ictonychinae and Mustelinae), badgers (Taxidiinae, Mellivorinae, Melinae, and Helictidinae), martens (Guloninae), minks (Mustelinae), and otters (Lutrinae). These mustelids are primarily found in the northern hemisphere, and terrestrial species in Japan include Japanese weasel ( *Mustela itatsi* , native), Siberian weasel ( *Mustela sibirica* , non‐native), common least weasel (*Mustela nivalis nivalis*, native), ermine ( *Mustela erminea* ssp., native), mink ( *Neovison vison* , non‐native), Japanese sable ( *Martes zibellina brachyura* , native), and Japanese badger ( *Meles anakuma* , native). Of these, *M. itatsi* , *M. sibirica, M. erminea*, and *N. vison* are distributed in areas south of Honshu, which are endemic for *T. callipaeda*, and there is a possibility that *T. callipaeda*‐infected individuals may be detected in the future. The pathogenicity of *T. callipaeda* to mustelids remains unclear due to the lack of pathological examination and insufficient observational data to determine its impact. In wild animals, infections are usually asymptomatic; however, severe infections have been reported to cause pruritus, secondary infections, and blindness (Bezerra‐Santos et al. 2022; Bertos et al. 2023). There is a need for further investigation into the pathogenicity of wild mustelids and their impact on conservation efforts.

**TABLE 1:** Reports of *Thelazia callipaeda* in wild mustelids.

Host			*T. callipaeda*			Reference
Subfamily	Species	Country	Positive/overall (prevalence, %)	Burden, min–max (mean)	Haplotype	
Guloninae	Beech marten ( *Martes foina* )	Italy	3/22 (13.6)	3–6 (5.0)	h1	Otranto et al. (2009)
		Portugal	1/—	1	h1	Seixas et al. (2018)
		Romania	1/13 (7.7)	1	h1	Ionică et al. (2019)
	Sable ( *Martes zibellina* )	Russia	28/492 (5.7)	1–6 (2.2)	nd	Odoevskaya et al. (2015)
	Japanese marten ( *Martes melampus melampus* )	Japan	1/—	11	h9, h10	This study
Melinae	European badger ( *Meles meles* )	Romania	1/55 (1.8)	33	h1	Ionică et al. (2019)
	Japanese badger ( *Meles anakuma* )	Japan	1/—	2	h9	Kitajima et al. (2024)

Abbreviation: nd, not done.

## Author Contributions

**Toshihiro Tokiwa:** conceptualization (lead), funding acquisition (lead), project administration (lead), visualization (lead), writing – original draft (lead), writing – review and editing (lead). **Kandai Doi:** conceptualization (supporting), investigation (equal), project administration (equal), resources (equal), writing – original draft (supporting), writing – review and editing (equal). **Ayaka Kitajima:** data curation (lead), investigation (equal).

## Conflicts of Interest

The authors declare no conflicts of interest.

## Data Availability

Nucleotide sequence data reported in this paper are available in the GenBank, EMBL, and DDBJ databases under the accession numbers: PV291673 and PV291674.
